# Costing electronic private sector malaria surveillance in the Greater Mekong Subregion

**DOI:** 10.1186/s12936-021-03727-w

**Published:** 2021-04-20

**Authors:** Ann Levin, Rebecca Potter, Kemi Tesfazghi, Saysana Phanalangsy, Phally Keo, Elijah Filip, Si Hein Phone, M. James Eliades

**Affiliations:** 1 Levin & Morgan LLC, Bethesda, MD USA; 2 University of Oslo, Oslo, Norway; 3 Population Services International, Vientiane, Laos; 4 Population Services International, Yangon, Myanmar; 5 Population Services International, Phnom Penh, Cambodia; 6 Clinton Health Access Initiative, Phnom Penh, Cambodia; 7 Asia Pacific Malaria Elimination Network, Singapore, Singapore; 8 Independent Consultant, New York, NY USA

**Keywords:** Malaria, Surveillance, Cost, Private sector

## Abstract

**Background:**

Private sector malaria programmes contribute to government-led malaria elimination strategies in Cambodia, Lao PDR, and Myanmar by increasing access to quality malaria services and surveillance data. However, reporting from private sector providers remains suboptimal in many settings. To support surveillance strengthening for elimination, a key programme strategy is to introduce electronic surveillance tools and systems to integrate private sector data with national systems, and enhance the use of data for decision-making. During 2013–2017, an electronic surveillance system based on open source software, District Health Information System 2 (DHIS2), was implemented as part of a private sector malaria case management and surveillance programme. The electronic surveillance system covered 16,000 private providers in Myanmar (electronic reporting conducted by 200 field officers with tablets), 710 in Cambodia (585 providers reporting through mobile app), and 432 in Laos (250 providers reporting through mobile app).

**Methods:**

The purpose of the study was to document the costs of introducing electronic surveillance systems and mobile reporting solutions in Cambodia, Lao PDR, and Myanmar, comparing the cost in different operational settings, the cost of introduction and maintenance over time, and assessing the affordability and financial sustainability of electronic surveillance. The data collection methods included extracting data from PSI’s financial and operational records, collecting data on prices and quantities of resources used, and interviewing key informants in each setting. The costing study used an ingredients-based approach and estimated both financial and economic costs.

**Results:**

Annual economic costs of electronic surveillance systems were $152,805 in Laos, $263,224 in Cambodia, and $1,310,912 in Myanmar. The annual economic cost per private provider surveilled was $82 in Myanmar, $371 in Cambodia, and $354 in Laos. Cost drivers varied depending on operational settings and number of private sector outlets covered in each country; whether purchased or personal mobile devices were used; and whether electronic (mobile) reporting was introduced at provider level or among field officers who support multiple providers for case reporting.

**Conclusion:**

The study found that electronic surveillance comprises about 0.5–1.5% of national malaria strategic plan cost and 7–21% of surveillance budgets and deemed to be affordable and financially sustainable.

**Supplementary Information:**

The online version contains supplementary material available at 10.1186/s12936-021-03727-w.

## Background

The World Health Organization (WHO) urges National Malaria Programmes to transform surveillance into a core intervention to drive progress from control to elimination [1]. Countries in the Greater Mekong Subregion (GMS) are updating national systems to support the requirements of surveillance in elimination settings. National Malaria Control Programmes (NMCPs) are considering whether and how to transition from aggregate, paper-based reporting systems to real-time, case-based electronic systems. The rapid growth of smart phone ownership and mobile coverage in the GMS [2] is also prompting NMCPs and malaria partners to integrate mobile reporting solutions into surveillance systems to increase the timeliness and granularity of case reporting.

Population Services International’s Greater Mekong Subregion Elimination of Malaria through Surveillance (GEMS) programme, funded by the Bill and Melinda Gates Foundation (BMGF), contributed to government-led malaria elimination strategies in Cambodia, Lao PDR, and Myanmar and Vietnam by developing electronic systems for private sector providers during the 2013–2018 period. In response to increasing demand for real-time case-based reporting and analysis, the GEMS programme designed and configured an electronic surveillance platform in DHIS2, developed open-source mobile applications for real-time reporting, and scaled mobile reporting tools across private sector networks. Malaria case data reported by PSI’s networks of private providers and captured through the GEMS surveillance platform were integrated into national systems at varying degrees of frequency and granularity, in accordance with NMCP reporting protocols and depending on the readiness of national surveillance systems. As a result of the intervention, the timeliness of private provider reporting a suspected or positive malaria case was reduced from a month to less than 24 h in Cambodia and Lao PDR. In Myanmar, the 16,000 private providers went from not reporting their malaria cases to reporting these monthly.

The study authors conducted cost analyses in parallel with programme implementation to provide information on the key cost drivers and assess affordability and financial sustainability of introducing and maintaining electronic surveillance approaches across three operational settings in Cambodia, Lao PDR, and Myanmar, as well as to estimate the comparative costs of approaches in different programmatic settings.

Cambodia, Lao PDR, and Myanmar have committed to achieving malaria elimination by 2030, although each country is at a different stage in the elimination pathway in terms of each country’s epidemiology and the readiness of surveillance and response systems [3–5]. Malaria caseloads and mortality rates continue to fall, and malaria transmission is becoming increasingly localized. Transmission hot spots are often concentrated in and around forested areas, affecting populations who live or work in these areas. As a result, many of the “last mile” malaria cases are expected to occur in remote areas, where access to public health services can be poor. Large proportions of malaria patients in the GMS seek and receive malaria treatment from the private sector. Proportions vary depending on key demographic indicators, such as living in urban or rural locations and wealth quintile, with estimates of up to 65% of the population seeks malaria care in the private sector in Myanmar and 75% in Cambodia [6]. In Lao PDR, a study in a malaria endemic districts showed that 77% of patients first sought care in the private sector [7]. Private sector malaria providers include formal health care providers such as clinics and pharmacies, as well as non-formal outlets, including drug shops, mobile drug vendors, and general retailers according to varying national policies.

The PSI GEMS project has been working with large networks of private sector outlets and providers—both formal and non-formal located in areas of transmission—to facilitate case reporting into national surveillance systems and ensure that NMCPs have access to complete data for evidence-based decision making. The project used DHIS2, an open-source health management information system adopted by more than 60 countries worldwide, as an electronic platform to collect, analyze, and report routine surveillance data from the private sector. Ministries of Health in Myanmar and Lao PDR also use DHIS2 as a national health management information system. The project designed, developed and scaled mobile reporting applications to improve timeliness of case-based reporting from private providers. These tools include PSI’s custom-developed Malaria Case Surveillance App and the University of Oslo’s generic, freely available DHIS2 mobile app. Both tools enable providers to submit case data to DHIS2 in near real-time through Android mobile devices.

The providers fill in data on the total number of persons tested for malaria, suspected malaria cases, suspected cases tested, and confirmed malaria cases reported by private providers. In Cambodia and Laos, the data are transmitted in near real-time while the data in Myanmar are sent monthly to the NMCPs.

Despite substantial investments in surveillance system strengthening, the evidence on affordability and feasibility of financially sustaining private sector surveillance in elimination settings is limited. Most studies on surveillance costs focus on other malaria programme enhancements such as improved diagnostic methods [8] or GIS systems [9]. The purpose of this study is to provide estimates on the affordability and financial sustainability of electronic surveillance interventions through an analysis and comparison of the costs in Cambodia, Lao PDR, and Myanmar. These insights will provide evidence to NMCPs and partners to inform national decisions on resource allocation and surveillance strengthening strategies.

## Methods

### Study area

The costing study was conducted in three countries: Cambodia (14 out of 25 provinces), Lao PDR (five out of 17 provinces), and Myanmar (nationwide) from 2017 to 2018. The electronic surveillance system covered 16,000 private malaria providers in Myanmar using 200 field officers to collect data with PSI-provided tablets; it covered 710 private providers in Cambodia, including 585 providers reporting through PSI-provided smartphones devices using PSI’s Malaria Case Surveillance App (limited to 585 due to budget considerations) and 125 providers using paper-based reporting; and it covered 432 providers in Lao PDR, with 250 private providers reporting through personal smartphone devices using PSI’s Malaria Case Surveillance App) and 182 using paper-based reporting.

The programme supported two commodities for case management—rapid diagnostic tests for testing, and artemisinin-based combination therapy (ACT) for treatment of malaria (when permitted by governments). The private providers were identified through annual mapping of private sector outlets.

In Myanmar, malaria providers included two types of private providers: (i) trained health providers (general practitioners and community-based health providers), and (ii) commercial outlets (e.g. general retailers, drug shops) (see Table 1). They were identified through annual routine mapping of the private sector.

**Table 1:** Providers authorized by governments to test and treat for malaria

Cambodia	Lao PDR	Myanmar
Health providers in clinics and cabinets Private worksites (e.g. plantations) with trained on-site Mobile Malaria Workers	Private providers in pharmacies and health clinics	Private general practitioner clinics General retailers, sundry shops and itinerant drug vendors Community-based health service providers

In Laos and Cambodia, only private health facilities and pharmacies are authorized to conduct malaria testing and treatment while grocery stores and general retailers are not allowed to sell anti-malarials. During the electronic surveillance introduction, network malaria providers in Laos included private providers (clinics and pharmacies), while in Cambodia, malaria providers were comprised of private providers (health clinics and single provider offices) and providers based on private worksites in high risk areas (e.g. plantations).

### Study design

A micro-costing approach (ingredients-based) [10] was applied to estimate the value of resources used in the surveillance interventions. The resources (*costing inputs*) used for every activity were identified and a unit cost attached to each resource. The list of activities was agreed upon through discussions with implementing and finance programme staff in the study countries and are shown in Table 2. Activities are not limited to the introduction of software itself; the list includes all activities that are required for reporting case data from private providers into the electronic system and generating automated analytic outputs.

**Table 2:** Costing activities

	Intervention category	Activities	Persons responsible for activity
Start-up costs	Introduction of electronic surveillance system (DHIS 2)	System design System configuration Piloting Training Design of surveillance bulletins	NGO/National/International staff^a^ Consultants (DHIS2)
	Introduction of mobile reporting	Mobile application design Mobile application development Piloting mobile applications Training providers on mobile reporting	National/international staff Consultants (DHIS2/Android)
Capital costs	Introduction of electronic surveillance system (DHIS 2)	Procurement of hardware: desktops	National staff
	Introduction of mobile reporting	Procurement of hardware: mobile devices	National staff
Recurrent costs	Electronic surveillance system (DHIS 2)	Data entry (centralized) System upgrades and maintenance Server hosting Supportive supervision of malaria providers Data quality assurance, monitoring and evaluation Production and dissemination of surveillance reports	NGO/National staff^b^ Consultants (system upgrades and maintenance)
	Mobile reporting	Mobile app upgrades and maintenance Mobile data packages	National staff Consultants (mobile app upgrades and maintenance)

^a^NGO staff assisted with system configuration

^b^Supervision is conducted by NGO workers

The costs of the private provider surveillance system has two components: (1) the electronic surveillance system that includes the design, configuration and maintenance of the DHIS 2 platform for malaria surveillance; and (2) mobile reporting that refers to the application design, application development, piloting applications, and training providers as well as data packages, upgrades and maintenance, refresher training and supervision.

The study perspective is the implementer and private provider’s (PSI’s GEMS project) and financial and economic costs were differentiated. Financial costs are monetary outlays spent by implementers on resources used for electronic surveillance interventions whereas economic costs include all costs, including opportunity costs (e.g. the value of private providers’ time spent in programme activities such as training and supervision as well such as donated goods and volunteer time). Both financial and economic costs include staff costs for the activities. However, the opportunity cost of private provider time spent in training and meetings was only estimated for economic costs and not financial costs since these were not paid for by the ‘payer’. The value of provider time was estimated using country-specific minimum wages multiplied by estimated number of hours spent on the activity. Note that the 2016 exchange rates used for the minimum wages were the following: 3990 Cambodia riels to USD $1, 8200 Laotian kip to US $1, and 1364 Myanmar kyat to US $1. It should be noted, however, that the providers in Cambodia and Lao PDR did benefit from the mobile data packages.

Activity costs were categorized as “start-up”, “capital” or “recurrent”. Start-up costs are one-time activities to prepare for the project such as initial training, system design, and mobile application design. In the case where capital costs (e.g. smartphones, tablets, desktops, computer servers) were used, these were amortized as part of the total cost estimates. Recurrent costs include the value of resources that last less than 1 year. For financial costs, straight-line depreciation was used (i.e. the capital costs were divided by the number of useful life years) while amortization and discounting was used in economic costs. Research costs were excluded from the estimation. The timing of the start-up activities differed among the three countries. The cost of these were converted to 2016 USD using the World Bank Consumer Price Index. The study team estimated total costs and cost per provider for the cost of the electronic surveillance system and cost of mobile reporting (Additional file 1: Table S1).

### Study timing and duration

Data collection took place between October 2018 and May 2019. Costs associated with the introduction and maintenance of surveillance interventions were included in the study based on the timing of implementing these interventions in each participating country. The study assumed an implementation period of 3 years to enable comparison across countries. The study team collected cost data for the period from October 2013 through September 2018 in Cambodia; from April 2016 through September 2018 in Lao PDR, and from November 2015 through September 2018 in Myanmar. Costs were derived by assigning values to resources utilized such as training and private provider time spent in training and monthly meetings, and quantities utilized. The data collection was informed in part by discussions with key informants from key stakeholders in each setting. The timing of the start-up costs differed by country—i.e. 2013–2016 in Cambodia, 2016 in Lao PDR, and 2015–2016 in Myanmar.

Since implementation took place over 3 years, costs were annualized with straight line depreciation for financial costs (dividing by 3) and amortization with a discount factor of 3% through dividing by an annualization factor (2.829) for economic costs.

### Sensitivity analysis

Sensitivity analysis was conducted for some parameters that are uncertain, specifically the discount rate, the value of private provider time spent on training and data entry on electronic and paper forms, and the percentage of time spent on supervision.

### Affordability and financial sustainability

To assess financial sustainability, the study team assessed the government’s ability to fund malaria surveillance by comparing the annual cost of electronic surveillance with the annual budget for implementing its national malaria strategic plans and surveillance. Data on the costs of national strategic plans were taken from the three countries so that the estimated costs of electronic surveillance could be compared with national annual budgets for surveillance and the malaria programme.

## Results

### Cambodia

In Cambodia, the project developed a custom mobile reporting app, the Malaria Case Surveillance App (MCS), for providers to be able to report cases directly to DHIS2 through a mobile smartphone device. The project procured and equipped 585 of 710 private providers with smartphones, trained providers to report cases through the mobile app, and provided monthly data packages to the private providers to support use of the app.

Table 3 shows the financial and economic costs for introducing electronic surveillance and mobile reporting in Cambodia during 3 years by cost category: start-up, capital or recurrent. Total financial costs for the Cambodian intervention were $434,968, while annual financial costs were $144,989. Total economic costs were $891,748 in total and 297,249 annually. The annual financial and economic costs per provider were $240 and $371, respectively.

**Table 3:** Financial and economic costs of Cambodia electronic surveillance intervention and provider mobile reporting intervention

Cost category	Total cost	%
Financial costs		
Start-up		
System design and configuration (DHIS2)	$52,933	12.1%
Piloting (DHIS2)	$11,545	2.3%
Training (DHIS2)	$7847	1.5%
MCS App design and development	$24,817	4.9%
MCS App piloting	$9475	1.9%
MCS App training	$8054	1.6%
Surveillance bulletin design	$2366	0.5%
Server set-up	$150	0.0%
Sub-total start-up	$120,616	23.6%
Capital		
Equipment (desktops)	$4800	0.9%
Equipment (mobile devices)	$112,155	21.9%
Sub-total capital	$116,955	22.9%
Recurrent		
DHIS2 maintenance and routine upgrades	$22,592	5.1%
DHIS2 software upgrades	–	0.0%
Data entry	$71,070	13.9%
Server hosting	$7620	1.5%
MCS App maintenance and routine upgrades	$32,182	6.3%
Procure electronic report: Monthly mobile data packages	$104,580	20.5%
Provider social and behavioural change communication (SBCC) and supervision	$35,569	7.0%
Sub-total recurrent	$273,612	53.5%
Total costs	$511,183	100%
Annual costs	$170,394	N/A
Annual cost per mobile reporting provider (n = 585 providers)	$291	N/A
Annual cost per provider (n = 710 providers)	$240	N/A
Economic costs		
Start-up costs		
System design (DHIS2)	$218,782	27.7%
Piloting (DHIS2)	$12,242	1.6%
Training (DHIS2)	$12,891	2.4%
MCS App design and development	$26,317	3.3%
MCS App piloting	$10,048	1.3%
MCS App training	$10,800	1.1%
Surveillance bulletin design	$2508	0.3%
Server set-up	$159	0.0%
Sub-total start-up	$297,703	37.7%
Capital		
Equipment (desktops)	$5090	0.6%
Equipment (mobile devices)	$118,934	15.1%
Sub-total capital	$124,024	15.7%
Recurrent		
DHIS2 maintenance and routine upgrades	$22,592	2.9%
DHIS2 software upgrades	$30,000	3.8%
Paper-based and electronic data entry	$110,498	14.0%
Server hosting	$7620	1.0%
MCS App maintenance and routine upgrades	$32,182	4.1%
Procure electronic report: monthly mobile data packages	$129,483	16.4%
Provider social and behavioural change communication (SBCC) and supervision	$35,569	4.5%
Sub-total recurrent	$367,944	46.6%
Total costs	$789,671	100%
Annual costs	$263,224	N/A
Annual cost per mobile reporting provider (n = 585 providers)	$450	N/A
Annual cost per provider (n = 710 providers)	$371	N/A

Economic costs included the estimated value of private provider time on training and reporting (electronic and paper) as well as the value of DHIS2 software, a global public good supported by the University of Oslo that makes the software freely available. It was estimated that supporting a new software for the system would cost $150,000 in in total or $50,000 per year; and routine maintenance upgrades to the software were valued at $30,000 total and $10,000 per year. The costs to the payer (financial costs) are higher for mobile reporting than for introduction of electronic surveillance with DHIS2. This is due to the cost of the mobile phones and the monthly data packages. However, both costs are similar for economic costs when the value of the ‘free’ DHIS2 software are taken into account.

**Fig. 1:**
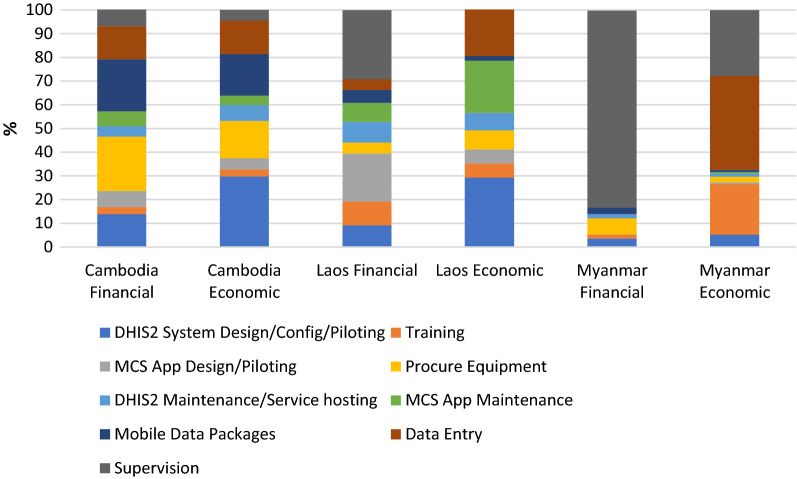
Financial cost of electronic surveillance and mobile reporting by category in Cambodia, Lao PDR, and Myanmar

The largest share of financial total costs was for recurrent costs (54%), due to the cost of the monthly mobile data packages and other costs of implementation, followed by similar shares for start-up activities and capital goods (24% and 23%, respectively). The largest share for economic costs was also for recurrent (48%), followed by startup (37%). Figure 1 shows bar chart for the financial and economic costs for electronic surveillance and mobile reporting in Cambodia, Lao PDR, and Myanmar.

### Lao electronic surveillance

Table 4 shows that total financial costs for the Lao intervention were $160,323, while annual costs were $53,441. Total economic costs were $458,414 while annual costs were $152,805. The annual financial and economic costs per provider were $124 and $354, respectively.

**Table 4:** Financial and economic costs of Lao PDR electronic surveillance intervention and provider mobile reporting intervention

Cost category	Total costs	%
Financial costs		
Start-up		
System design and configuration (DHIS2)	$5768	5.5%
Piloting (DHIS2)	$595	0.4%
Training (DHIS2)	$7473	4.7%
Server set-up	$150	0.1%
Surveillance bulletin design	$5107	3.2%
MCS App design and development	$27,362	16.9%
MCS App piloting	$5659	3.5%
MCS App training for providers	$8281	5.2%
Sub-total start-up	$63,250	39.5%
Capital		
Hardware (desktops)	$2400	1.5%
Equipment (tablets for field officers)	$4800	3.0%
Sub-total capital	$7200	4.5%
Recurrent		
DHIS2 maintenance and routine upgrades	$8245	5.1%
Server hosting	$5976	3.7%
MCS App maintenance and routine upgrades	$12,877	8.0%
Monthly mobile data packages	$8640	5.4%
Paper-based data entry	$7445	4.6%
Provider BCC and supervision	$44,490	28.0%
Monitoring and evaluation	$1750	1.1%
Sub-total recurrent	$89,873	56.1%
Total costs	$160,323	100%
Annual costs	$53,441	N/A
Annual cost per private provider (n = 432)	$124	N/A
Annual cost per private provider reporting by mobile (n = 250)	$214	N/A
Economic costs		
Startup cost		
System design and configuration (DHIS2)	$168,446	36.8%
Piloting (DHIS2)	$631	0.1%
Training (DHIS2)	$13,243	2.9%
Server set-up	$159	0.0%
Surveillance bulletin design	$5415	1.2%
MCS App design and development	$28,781	6.2%
MCS App piloting	$6001	1.3%
MCS App training for providers	$10,156	2.2%
Sub-total startup	$232,833	50.8%
Capital		
Hardware (desktops)	$2545	0.6%
Equipment (tablets for field officers)	$5090	1.1%
Equipment (provider smartphones)	$39,767	8.7%
Sub-total capital (annual)	$47,402	10.3%
Recurrent		
DHIS2 maintenance and routine upgrades	$8245	1.8%
DHIS2 software upgrades	$30,000	6.5%
Server hosting	$5976	1.3%
MCS App maintenance and routine upgrades	$12,877	2.8%
Monthly mobile data packages	$12,177	2.7%
Paper-based and electronic data entry	$62,215	13.6%
Provider BCC and supervision	$44,940	9.8%
Monitoring and evaluation	$1750	0.4%
Sub-total recurrent	$178,179	38.9%
Total costs	$458,414	100%
Annual costs	$152,805	N/A
Annual cost per private provider (n = 432)	$354	N/A
Annual cost per private provider reporting by mobile (n = 250)	$611	N/A

Economic costs also accounted for private provider time, the value of the DHIS2 software and the estimated cost of providers’ personal mobile devices. For financial costs, the largest share of total costs was for recurrent costs (56%) due to the high cost of supervision (28%). Among economic costs, start-up costs had the highest share (51%) due to the inclusion of the value of DHIS2 software (39%). Similar to Cambodia, the financial cost of mobile reporting is higher than that of electronic surveillance.

### Myanmar electronic surveillance

Table 5 shows the financial and economic costs of introducing electronic surveillance and mobile reporting in Myanmar. The mobile reporting model employed in Myanmar is different from Cambodia and Lao PDR since 200 field officers employed by PSI Myanmar reported electronically to DHIS2 with tablets when they visited providers monthly rather than providers using smartphones to report directly. Providers maintained paper records for primary reporting. Further, PSI Myanmar did not design or develop a customized mobile application for reporting. Instead, PSI Myanmar used a free generic, open-source DHIS2 reporting app.

**Table 5:** Financial and economic costs of Myanmar electronic surveillance intervention and mobile reporting intervention among field-based supervisors

Cost category	Total cost	%
Financial costs		
Start-up		
System design and configuration (DHIS2)	$39,363	3.0%
Piloting (DHIS2)	$4832	0.4%
Training (DHIS2)	$9361	0.7%
Server setup	$300	0.0%
Surveillance bulletin design	$2443	0.2%
App piloting	$4178	0.3%
App training for field officers	$12,672	1.0%
Sub-total start-up	$73,149	5.6%
Capital		
Tablets (for mobile reporting)	$80,200	6.1%
Hardware (desktops)	$9600	0.7%
Sub-total capital (annual)	$89,800	6.8%
Recurrent		
DHIS2 maintenance and routine upgrades	$10,070	0.8%
Server hosting	$13,680	1.0%
Provider social and behaviour change communication and supervision	$1,093,680	83.1%
Mobile data packages	$36,000	2.7%
Sub-total recurrent	$1,153,430	87.6%
Total costs	$1,316,379	100%
Annual costs	$437,954	N/A
Cost per provider (16,000 providers)	$27	N/A
Economic costs		
Startup costs		
System design and configuration (DHIS2)	$200,809	5.1%
Piloting (DHIS2)	$5124	0.1%
Training (DHIS2)	$875,760	22.0%
Server setup	$318	0.0%
Surveillance bulletin design	$2590	0.1%
App piloting	$4430	0.1%
App training for field officers	$13,438	0.3%
App development	$27,549	0.7%
Sub-total start-up	$1,130,019	28.3%
Capital		
Tablets (for mobile reporting)	$85,048	2.1%
Hardware (desktops)	$10,180	0.3%
Sub-total capital (annual)	$95,228	2.4%
Recurrent		
DHIS2 maintenance and routine upgrades	$40,070	1.0%
Paper and electronic data entry	$1,555,200	39.0%
Server hosting	$13,680	0.3%
Provider social and behaviour change communication and supervision	$1,093,680	27.4%
Mobile data packages	$38,244	1.0%
App maintenance	$22,500	0.6%
Sub-total recurrent	$2,763,374	69.3%
Total costs	$3,939,268	100%
Annual costs	$1,310,912	N/A
Cost per provider (16,000 providers)	$82	N/A

Total financial costs for the Myanmar intervention were $1,316,379, while annual financial costs were $437,954. Total economic costs were $3,988,621 and $1,310,357 annualized. Economic costs also accounted for the value of private provider time and DHIS2 software as in the other two countries. The economic cost of the custom app was $27,549, with no financial costs incurred by the programme for development. The annual financial and economic costs per provider were $27 and $82, respectively.

Recurrent costs represented 88% of the total share of financial costs. The cost driver was monthly supportive supervision to providers for capturing surveillance data from paper records. For economic costs, recurrent costs also had the largest share of total costs (69%) due to the value of private provider time spent on data entry and cost of supervision.

### Sensitivity analysis

Table 6 shows the impact of varying variables with uncertainty on annual economic cost: discount rate, value of provider time, and % supervisor time spent on visits. The impact is greatest when the value of provider time is varied.

**Table 6:** Annual economic costs (000 s USD) of electronic surveillance when key factors are varied

	Discount rate		Value of provider time		% supervisor time spent on visits	
	0%	5%	Minimum wage	General practitioner/clerk salary	− 25%	+ 25%
Cambodia	$255	$277	$267	$310	$262	$273
Lao PDR	$148	$156	$153	$202	$149	$158
Myanmar	$3863	$3978	$1311	$2536	$1220	$1402

### Comparison of electronic surveillance costs in Cambodia, Laos, and Myanmar

Cost structure varied across the three countries depending on the cost drivers, the size of the networks and the manner in which electronic reporting was implemented, i.e. individual provider level in Cambodia and Lao PDR and supervisor level in Myanmar. Myanmar’s coverage was national with a large number of providers covered (16,000) that resulted in a high annual economic cost ($1,310,912), primarily due to the cost of supervision, but a low annual cost-per-provider covered ($82) (Table 4). The numbers of providers covered in Lao PDR was much smaller (n = 432), leading to a lower annual economic cost ($152,805), but a higher cost-per-provider ($354) (Table 3). Cambodia had a network size (710) closer to Lao PDR’s and thus a similar annual economic cost-per-provider covered ($371). However, because the project in Cambodia developed the mobile app and purchased smartphones and data packages for all the providers, the annual economic costs were higher ($263,224) (Table 5). Neither Myanmar or Lao PDR developed a mobile app or purchased smartphones for their providers. Thus, supervision was the primary cost driver for financial cost.

Economic costs were compared to the annual national malaria strategic plans’ (NMSP) budgets in all three countries, and to the proportion specifically budgeted for surveillance in Lao PDR and Myanmar (there was no surveillance budget in the Cambodian NMSP). The total annual cost of PSI’s electronic surveillance intervention in the three countries comprised 0.5–1.5% of the total annual NMSP budget, and 6.8–20.5% of the total NMSP surveillance budget in Lao PDR and Myanmar (Table 7).

**Table 7:** Comparisons of estimated annual costs of electronic surveillance with National Malaria Program

	Economic cost of electronic surveillance	Estimated annual costs national malaria strategic plan	Estimated cost of surveillance in NMSP	% Electronic surveillance to annual program budget	% Electronic surveillance to estimated surveillance budget
Cambodia	$263,224 (14 out of 25 provinces)	$50,354,592	N/A	0.5	N/A
Lao PDR	$152,805 (5 out of 17 provinces)	$14,557,696	$2,260,000	1.0	6.8%
Myanmar	$1,310,912 (entire country)	$92,600,000	$6,400,000	1.5	20.5%

It should be noted that Myanmar used a different model of electronic surveillance (tablets with monthly reporting by NGO workers) than in Cambodia and Lao PDR where smartphones were used

## Discussion

Surveillance systems are a core intervention in malaria elimination settings [6]. Case-level data is needed from all health points of care that are diagnosing and treating malaria in order to properly estimate and allocate resources, target interventions, and implement elimination protocols. In many malaria endemic countries, private sector providers perform a significant proportion of case management and should be included in surveillance activities. Yet there is little evidence for national programmes to accurately cost the inclusion of private sector providers in malaria surveillance systems in elimination settings. While one other study investigated the cost of setting up a spatial decision support system for malaria elimination [9] and others have estimated the costs for other services [11], no others have focused on the costs of surveillance of private sector services. The costing of electronic surveillance systems to capture private sector malaria case data in Cambodia, Lao PDR, and Myanmar can provide a template for national programmes on elements to consider when costing out such a system, the main cost drivers, and how to implement in the most cost-effective way in different operational settings to promote affordability and financial sustainability.

Total annual economic costs of introducing electronic surveillance and mobile reporting were $152,805 in Lao PDR, $263,224 in Cambodia, and $1,310,912 in Myanmar. The financial cost drivers were recurrent in all three countries (mobile monthly packages in Cambodia and supervision in Lao PDR and Myanmar). For economic costs, the cost drivers were startup costs in Cambodia and Lao PDR (system design) since donated software and hardware were included. For Myanmar which had fewer hardware costs, the economic cost driver was supervision, a recurrent cost.

In Cambodia and Lao PDR, providers could report directly into the electronic surveillance system and there was less need for monthly data collection. Thus, supervision only accounted for 5% and 10% of the total annual economic costs in Cambodia and Lao PDR, respectively. While the network had similar sizes in Cambodia and Lao PDR (432 and 710, respectively), the providers in Lao PDR used their own smartphones and, as a result, a higher proportion of the total annual economic costs went towards start-up costs.

Myanmar had a lower cost per provider reporting malaria case data since it employed field officers to visit clinics monthly and record the data in tablets. This approach could be particularly useful in sub-national areas that have already developed a supportive supervision model and where it is geographically feasible to visit providers at least monthly. While this increases the proportion of total annual economic cost spent on supervision (69% in Myanmar), its costs were much lower due to the reduced need for mobile phones and monthly mobile data packages. However, such a system impacts timeliness, with reporting only occurring periodically rather than in real-time. Alternative solutions can be considered as caseloads drop. As sub-national areas approach elimination, field officers could be replaced or complemented with a system that targets smartphone purchase for higher case-load providers. Since smartphone penetration was already estimated to be 80% in 2019 [12], this type of reporting will be increasingly more feasible. For providers with few cases, a simple phone call to field officers could be utilized to complete required reporting and initiate malaria elimination surveillance protocols. While the PSI GEMS project was an NGO-supported, malaria-focused surveillance programme, NMPs may identify other efficiencies, such as incorporating supervision and data collection for private sector providers into existing public sector implementation programmes and including private sector providers when training public sector providers.

Some lessons learned about cost-efficiencies can be found from comparing the three settings. In Cambodia, smartphones were purchased for most providers and this made up 15% of the total annual economic cost of the intervention with an additional 16% spent on mobile-date packages. In Lao PDR where the 250 providers that were mobile reporting used their own smartphones, the cost was half that proportion, and in Myanmar, where 200 tablets were purchased for supervisors to then collect and send the data from the 16,000 providers, the costs were just above 2% of the total. Implementing mobile-based reporting where smartphone ownership is high may limit the costs of purchasing new phones and could be considered where provider networks are large. In settings where personal ownership of smartphones is low and Internet connectivity is variable, a model such as Myanmar’s could be used.

There are lessons in the three study countries that NMPs can consider when balancing the need to purchase smartphones and mobile-data packages, and the costs of supervision and data collection based on the size of the network. Rather than waiting until national caseloads are approaching elimination levels, high-burden countries could begin to explore options early on in preparation for an elimination-ready surveillance system. This system will need to capture case data from all types of providers, perhaps by focusing on areas of relatively lower transmission or other practical considerations that can eventually inform national elimination strategy. In the future, as internet connectivity is improved, governments can also consider having providers use already existing social media apps to send data on malaria cases.

In order to improve sustainability of the intervention and progress towards malaria elimination, it will be important for countries to gradually take over the costs of the intervention, including supervision of private providers.

All three countries are lower-middle income countries and should start absorbing programmatic costs. Recurrent financial costs, costs that governments will need to assume over time, were larger than capital and start-up costs in all three countries (47–88%). NMPs will need to ensure that recurrent costs are budgeted for over time. These may include costs associated with maintenance, upgrades, hosting, mobile data packages, and regular visits for data quality checks, supervision, and for providers without the ability to report themselves through mobile apps, to collect and report data.

Electronic surveillance appears to be affordable in the three study countries if one assumes that the cost estimated for implementing the national malaria strategic plans are accurate. The cost of introducing electronic surveillance is only a small percentage (0.5–1.5%) of the total malaria programme budgets, so that even when considering the addition of costs associated with implementation of public sector surveillance, inclusion of private sector providers is very likely feasible. Illustrative of that point in Lao PDR and Myanmar are the percentage of the electronic surveillance costs calculated in this study to the estimated surveillance budget in the NMSP (Cambodia’s NMSP did not have a specific budget line for surveillance), 6% and 8% respectively. Myanmar demonstrates the potential for countries with very large, nationally active, private sectors to implement an affordable electronic surveillance system.

The development of health information architecture is also an important factor in developing surveillance systems. The lack of adequate health information architecture to capture high quality case-based data has been cited as a gap in the performance of surveillance systems [13]. In the three study countries, the proportion of total economic costs to start-up an electronic DHIS2 ranged between 28 and 51% of total costs and included system design, configuration, server set-up, and piloting and training of personnel on DHIS2. Again, recurrent annual costs are important to consider, and in the three study countries ranged between 39 and 69% of the total annual economic costs. Budgeting to develop these systems now while in a control-phase with an eye to real-time, case-based reporting could potentially facilitate the transition to surveillance as a core intervention that goes hand-in-hand with case management.

The study had some limitations. The authors were unable to assess the benefits of the electronic surveillance. In addition, other factors that may affect the cost of supervision, such as difficulty of travel and proximity of supervisors to the providers they were overseeing, were not taken into account for this analysis. The study also compared two types of models of electronic surveillance that were not completely comparable—i.e. use of mobile phones in Cambodia and Lao PDR to enable real-time reporting of malaria cases while electronic tablets were used for monthly reporting of cases by field officers in Myanmar.

Another limitation is that the PSI staff have salaries that are higher than government salaries in the countries where they are working, i.e. two to six times the government salaries. Thus, the costing is overestimating the cost of the intervention if the governments were to take over the supervision role. In addition, some costs of activities such as the initial mapping of the private sector outlets were not included in the analysis.

## Conclusion

Costing the PSI electronic surveillance system in three study countries in the Greater Mekong Sub-region with unique operational elements can be used to inform other countries’ decision-making on planning for an electronic surveillance system that captures private sector data into national surveillance systems as per WHO recommendations. Recurrent annual cost estimates are particularly useful considering the long tail of elimination and the need to maintain surveillance activities until elimination is achieved and to prevent re-introduction.

## Supplementary Information

**Additional file 1: Table S1.** Description of cost categories.

## Data Availability

The datasets used and/or analysed during the current study are available from the corresponding author on reasonable request.

## Abbreviations

BMGF: Bill and Melinda Gates Foundation
DHIS2: District Health Information System 2
GEMS: Greater Mekong Subregion Elimination of Malaria through Surveillance
GMS: Greater Mekong Subregion
Lao PDR: Lao People’s Democratic Republic
NMCP: National Malaria Control Programmes
PSI: Population Services International
SBCC: Social and Behaviour Change Communication
WHO: World Health Organization
