# Supplementary material for: Costing electronic private sector malaria surveillance in the Greater Mekong Subregion
Source: Malar J. 2021 Apr 20;20:192. doi: 10.1186/s12936-021-03727-w (PMC8056544; doi:10.1186/s12936-021-03727-w)
Supplement: Supplementary file 1 — Additional file 1: Table S1. Description of cost categories. [file 12936_2021_3727_MOESM1_ESM.docx]

**Additional file**

**Table S1. Description of cost categories**

| **Cost category** | **Description** |
| --- | --- |
| **DHIS2 System design** | This activity includes M&E/MIS assessments, requirements gathering, and design documentation. This activity includes the time of consultants, the M&E Advisor, and local staff in designing the DHIS2 system. |
| **DHIS2 System Configuration** | This activity includes training of configuration consultants, configuration, project management and technical supervision. It includes the time of consultants, program managers, and local staff. |
| **DHIS2 Piloting** | This activity includes the time of local staff to pilot DHIS2 – i.e. MIS coordinator, data entry clerk, MIS manager, and MIS coordinator. |
| **Training DHIS2** | This activity includes DHIS2 regional trainings, MIS/system administrative training, data entry training, end user training/analytics trainings, partner/government training, and curriculum development. It includes the time of consultants, local staff, and DHIS2 consultants. |
| **DHIS2 Maintenance** | This activity includes testing for DHIS2 upgrades, DHIS2 upgrade documentation, and routine system support. It includes time of consultants, program managers, and local staff. |
| **Procurement Hardware** | This activity includes server setup, server hosting, hardware (laptops), and internet services. |
| **Surveillance Bulletin Design** | This activity includes dashboard & surveillance bulleting design and dissemination of surveillance bulletins. It includes the time of consultants, local staff, and the M&E advisor. |
| **App Design** | This activity includes requirements gathering and documentation, graphic design, and translation. It includes the time of local staff and consultants. |
| **App Development** | This activity includes coding, bug testing and fixing, project management, and technical supervision. It includes the time of consultants and local staff. |
| **Piloting App** | This activity includes bug fixing and field testing. It includes the time of consultants and local staff. |
| **Training App** | This activity includes internal staff training, provider training for reporting on apps, training of partners/government partners, training curriculum development and phone setup. It includes the time of consultants and local staff. |
| **App Maintenance** | This activity includes routine bug fixing, upgrades for compatibility with new DHIS2 version, app upgrades for new functionality/new requirements, specification development, and data signal coverage assessment. It includes consultants and local staff. |
| **Procurement electronic report** | This activity includes server setup, server hosting (cloud based-BAO), procurement of mobile phones and tablets, and monthly top-ups. It includes the time of local staff and the cost of smart phones, sim cards, tablets, and monthly top ups. |
| **Provider Electronic and Paper Data Entry** | This activity includes estimation of the time spent by private providers on electronic and paper based data entry. It involves estimating the value of this time by multiplying the average number of hours spent on data entry by the shadow wage of the private providers. |
| **Supervision** | This activity involves the supervision by NGO staff of private providers. It involves the time and per diem of the NGO staff and fuel for supervision. |
